# The costs of subsidies and externalities of economic activities driving nature decline

**DOI:** 10.1007/s13280-025-02147-3

**Published:** 2025-02-28

**Authors:** Victoria Reyes-García, Sebastian Villasante, Karina Benessaiah, Ram Pandit, Arun Agrawal, Joachim Claudet, Lucas A. Garibaldi, Mulako Kabisa, Laura Pereira, Yves Zinngrebe

**Affiliations:** 1 Institut de Ciència i Tecnologia Ambientals, Universitat Autònoma de Barcelona (ICTA-UAB), 08193 Cerdanyola del Vallès, Barcelona, Spain; 2 Institució Catalana de Recerca i Estudis Avançats (ICREA), 08010 Barcelona, Spain; 3 Departament d’Antropologia Social i Cultural, Universitat Autònoma de Barcelona, 08193 Cerdanyola del Valles, Barcelona, Spain; 4 CRETUS, EqualSea Lab, Department of Applied Economics, University de Santiago de Compostela, 15782 A Coruña, Spain; 5 Xunta de Galicia, Spain; 6 Department of Geography, Environment and Geomatics, University of Guelph, Guelph, ON Canada; 7 Centre for Environmental Economics and Policy, UWA School of Agriculture and Environment, The University of Western Australia, 35 Striling Highway, Crawley, Perth, WA 6009 Australia; 8 Global Center for Food, Land and Water Resources, Research Faculty of Agriculture, Hokkaido University, Kita 9, Nishi 10, Kita-ku, Sapporo, Hokkaido 060-8589 Japan; 9 The Western Australian Biodiversity Science Institute (WABSI), Perth, Australia; 10 School for Environment and Sustainability, University of Michigan, Ann Arbor, USA; 11 National Center for Scientific Research, PSL Université Paris, CRIOBE, Maison de l’Océan, 195 rue Saint-Jacques, 75005 Paris, France; 12 Instituto de Investigaciones en Recursos Naturales, Agroecología y Desarrollo Rural (IRNAD), Universidad Nacional de Río Negro, Mitre 630, 8400 San Carlos de Bariloche, Río Negro Argentina; 13 Consejo Nacional de Investigaciones Científicas y Técnicas, San Carlos de Bariloche, Río Negro Argentina; 14 Global Change Institute, University of the Witwatersrand, Private Bag 3, WITS, Johannesburg, 2050 South Africa; 15 Stockholm Resilience Centre, Stockholm University, Stockholm, Sweden; 16 Helmholtz Centre for Environmental Research – UFZ, Permoserstr 15, 04318 Leipzig, Germany; 17 Center for Cross-Disciplinary Research in Environmental Technologies (CRETUS), Av Rua Constantino s/n, 15782 Santiago de Compostela, Spain; 18 Keough School of Global Affairs, University of Notre Dame, Notre Dame, IN USA; 19 Johannesburg, South Africa

**Keywords:** Biodiversity, Environmentally harmful subsidies, Externality, Subsidy reform, Sustainable finance, Transformative change

## Abstract

Economic sectors that drive nature decline are heavily subsidized and produce large environmental externalities. Calls are increasing to reform or eliminate subsidies and internalize the environmental costs of these sectors. We compile data on subsidies and externalities across six sectors driving biodiversity loss—agriculture, fossil fuels, forestry, infrastructure, fisheries and aquaculture, and mining. The most updated estimates suggest that subsidies to these sectors total between US$1.7 and US$3.2 trillion annually, while environmental externalities range between US$10.5 and US$22.6 trillion annually. Moreover, data gaps suggest that these figures underestimate the global magnitude of subsidies and externalities. We discuss the need and opportunities of building a baseline to account for the costs of subsidies and externalities of economic activities driving nature decline. A better understanding of the complexity, size, design, and effects of subsidies and externalities of such economic sectors could facilitate and expedite discussions to strengthen multilateral rules for their reform.

**Supplementary Information:**

The online version contains supplementary material available at 10.1007/s13280-025-02147-3.

## Introduction

Human wellbeing and economic activities are inherently dependent on nature. Nature enables food production, regulates climate, supports the hydrological and carbon cycles, and contributes to human health (Díaz et al. 2019; Parmesan et al. 2023). More than half of global GDP is considered to be moderately or highly dependent on nature and its services (WEF 2020). And yet, humans are driving a pervasive decline of life on Earth (Díaz et al. 2019). Biodiversity loss and nature’s decline threat human wellbeing and create material risks for all nations and economic sectors. For example, biodiversity loss and nature’s decline could lower United Kingdom’s GDP by 6 to 12% than otherwise by 2030 under different scenarios (Avery et al. 2024). The World Bank Group estimates that if key nature’s contributions to people—such as pollination, marine fishing, and timber sourcing from native forests—are severely impacted, the global GDP could decline by US$2.7 trillion by 2030 (Johnson et al. 2021).

In this context, governments, international organizations, civil society, and some actors in the corporate sector agree that systemic change is urgently needed to protect biodiversity and restore ecosystems (Leclère et al. 2020; Tickner et al. 2020; Irvine-Broque and Dempsey 2023; Obura 2023). Calls for transformative change explicitly acknowledge the need for systemic change in the economic sectors most responsible for biodiversity loss and nature decline (O’Brien et al. 2024). Main direct drivers of nature decline—i.e. land- and sea-use change, direct exploitation of nature, climate change, pollution, and invasive alien species—are directly coupled to the agriculture, fossil fuels, infrastructure, forestry, fisheries, and mining sectors (Díaz et al. 2019). Currently, these economic sectors are heavily subsidized, with public investment in these sectors growing (Coady et al. 2017; FOLU 2019; Dempsey et al. 2020; Irvine-Broque and Dempsey 2023). For example, it is reported that the total amount of subsidies in the fossil fuel sector has witnessed a US$1.1 trillion increase from US$5.9 trillion in 2020 to US$7 trillion in 2022, expressed in 2021 US$ (Black et al. 2023) and the UNEP calculated that, since 2021, the total public funding for subsidies to sectors that drive biodiversity loss and nature decline has increased by 55% (UNEP 2023). In a recent update of their previous 2022 estimations, Koplow and Steenblik (2024) also report an increase in subsidies to economic activities that drive nature’s decline of roughly US$800 trillion (US$570 billion net of inflation) in two years. Compared to previous estimates, the increase is partly explained by the inclusion of estimates for non-energy mining and plastic production, but also by the increasing number of subsidies, particularly to fossil fuels.

While subsidies to these sectors could be directed to support activities that protect or restore nature (e.g. reforestation, pesticide-free agriculture, sustainable fisheries management), the current global subsidy scheme mostly incentivizes activities that accelerate the exploitation of the biosphere (Coady et al. 2017; Cottrell 2022; Koplow and Steenblik 2022; Segerson et al. 2024). Moreover, the economic sectors most responsible for nature decline not only benefit from government subsidies, but also from the omission of the full environmental costs of production in market prices. This leads to environmental externalities—unaccounted—for environmental impacts, such as pollution and habitat loss, that impose costs on society without being reflected in the price of goods and services (Coady et al. 2017; FOLU 2019). For example, industrial agriculture generates greenhouse gas emissions, water pollution, or soil degradation, but the costs of those impacts are not absorbed by the agricultural sector (FOLU 2019).

**Fig. 1:**
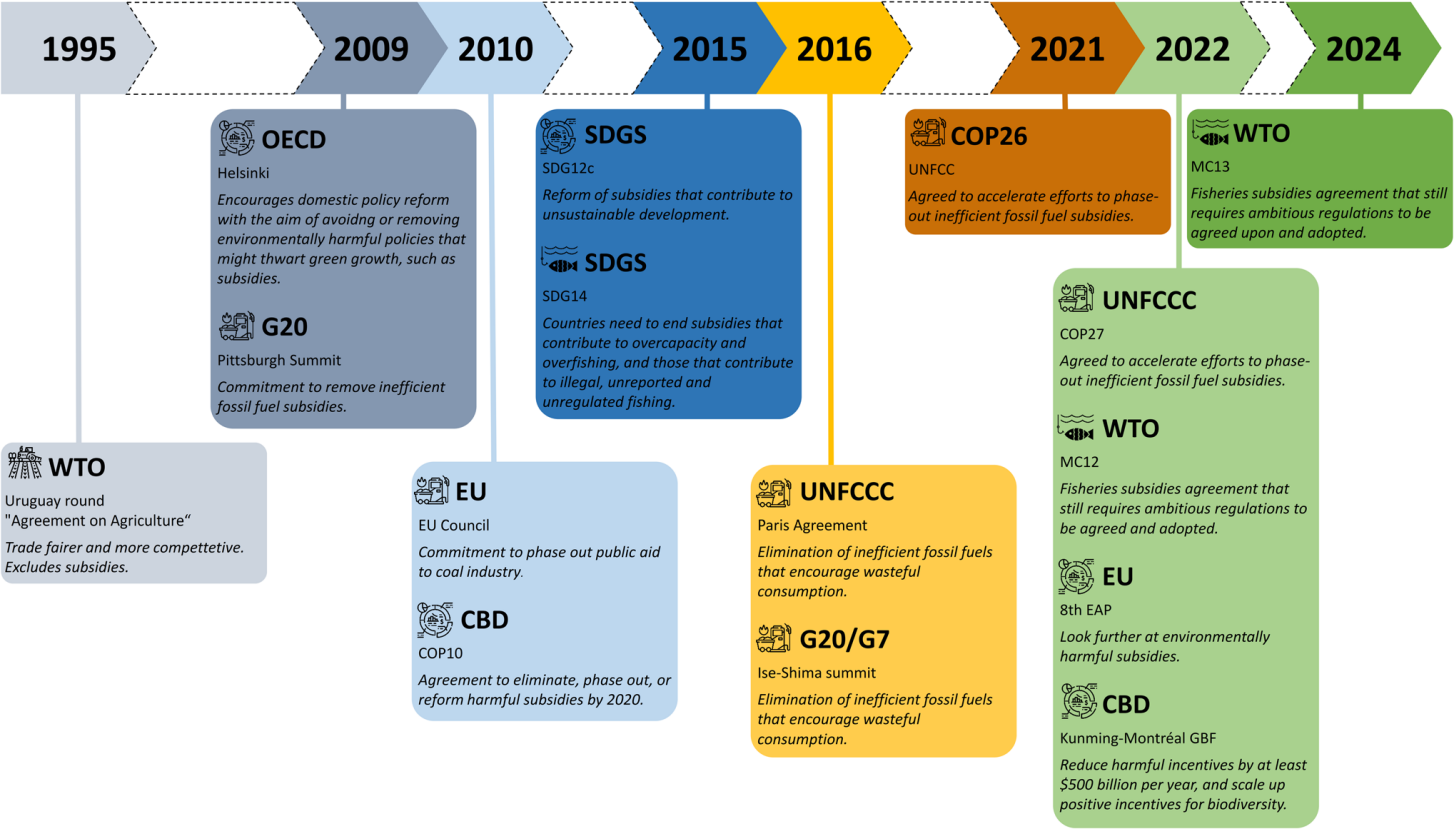
Temporal sketch showing major international efforts to reform environmentally harmful subsidies

In this context, the reform of sectors driving nature decline, including actions such as the elimination, phasing out, or reform of subsidies to these sectors and the internationalization of their externalities, has been in the international agenda for more than two decades, with calls intensifying recently (Fig. 1). For example, the Convention on Biological Diversity (CBD) recently identified the “reduction of harmful subsidies by at least US$500 billion per year and scale up positive incentives for biodiversity by 2030” as one of 23 targets necessary to achieve the 2050 vision for living in harmony with nature (COP15 2022). Calls have also been made to internalize externalities, by—for example—implementing consumption taxes (Black et al. 2023). Importantly, the reform of economic sectors driving nature decline requires a baseline that identifies the current magnitude of subsidies and externalities.

While the reporting of subsidies remains voluntary, several international organizations have started efforts to compile data on government subsidies across various economic sectors. For example, the Organisation for Economic Co-operation and Development (OECD) and the World Bank collect information on subsidies related to energy, agriculture, fisheries, and industry, while the Food and Agriculture Organization (FAO) focuses on subsidies in agriculture, fisheries, and forestry (Table S1). However, most of these organizations concentrate on specific sectors or operate within a limited regional scope. As a result, efforts to provide a comprehensive, global overview of subsidies remain scarce.

In this *Perspective* paper, we present results of our efforts to compile updated information on subsidies and externalities across six economic sectors directly associated with nature decline (i.e. agriculture, fossil fuel, infrastructures, forestry, fisheries-aquaculture, and mineral mining). Our review is based on a thorough compilation of data from secondary and tertiary sources, prioritizing the most comprehensive and up-to-date information available for each sector. We draw from a wide range of sources, including peer-reviewed and grey literature, national budgets, websites, databases, and other relevant materials (Table S1). To offer a comprehensive view of the relative value of subsidies across sectors, we aggregate data from the various sectors. Beyond presenting the most recent estimates, we also evaluate the current state of available data, identify methodological and data gaps, and discuss key considerations for establishing a robust baseline.

## Accounting for the costs

**Fig. 2:**
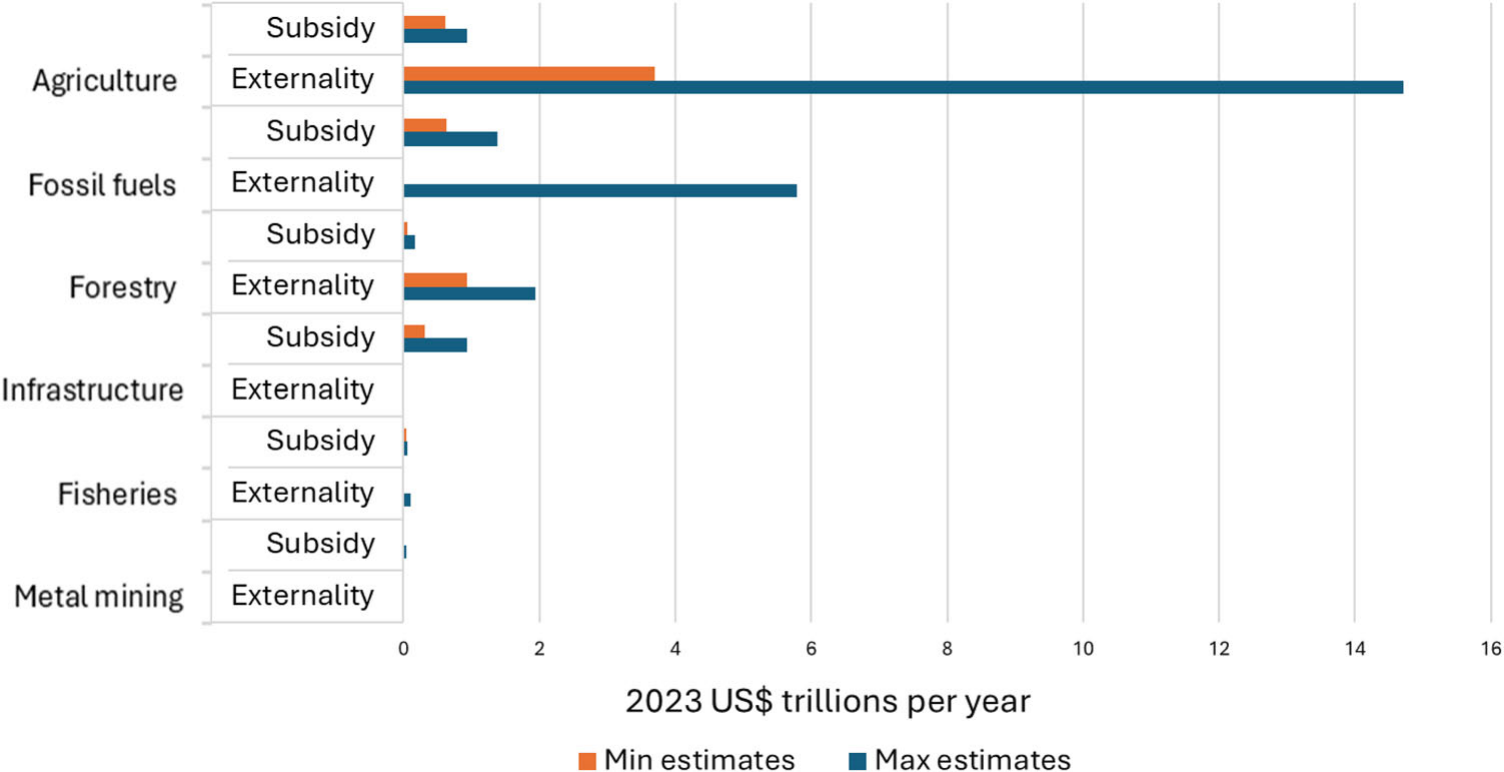
Bar chart showing estimated annual costs of subsidies and externalities for six economic sectors, expressed in 2023 US$. To construct the figure, estimates drawn from different sources and years were converted to 2023 US$ using inflation adjustments based on the U.S. Consumer Price Index for the corresponding data years (see Supplementary file1)

Figure 2 provides a compilation of updated data on subsidies and externalities across six economic sectors that are key drivers of nature decline.

### Agriculture

Agriculture, including crops and livestock, has led to large environmental pressures worldwide, affecting soil, freshwater, and forest preservation. Agriculture is also associated with land and marine biodiversity decline, as well as to greenhouse gas emissions (Henderson and Lankoski 2019; DeBoe 2020; Gautam et al. 2022; Damania et al. 2023). Food production is a major driver of environmental change, impacting six out of the nine planetary boundaries (Gordon et al. 2017). These impacts—ranging from inorganic fertilizers causing runoff into water systems through to methane emissions from livestock—have resulted largely from the intensification of agricultural production since 1961 (Gordon et al. 2017). Furthermore, agricultural land-use change is the greatest current threat to biodiversity, and the probable need for future agricultural expansion means that this land-use change will remain a major threat to biodiversity for the foreseeable future (Kehoe et al. 2017; Ortiz et al. 2021). Other concerning impacts lie in the interaction between historical climate warming and intensive agriculture, which is associated with reductions of almost 50% in the abundance and 27% in the number of species within insect assemblages relative to those in less-disturbed habitats with lower rates of historical climate warming (Outhwaite et al. 2022). At the same time, while industrial agriculture has impacted the environment heavily, it has not provided the necessary reduction in stunting or undernutrition, oftentimes driving other health-related impacts like malnutrition and obesity (Gordon et al. 2017; Willett et al. 2019).

Agriculture is one of the economic sectors receiving the largest subsidies (Fig. 2). Subsidies to agriculture include measures affecting farm gate prices, monetary transfers to farmers, and public expenditure and investment in goods and services that benefit the sector. Total estimates of explicit agriculture subsidies range from US$610 billion/year (Koplow and Steenblik 2024) to US$851 billion/year for OECD countries, with a 2.5-fold increase in 2020–22 compared to 2000–02 (OECD 2023). Damania and colleagues (2023) estimate agriculture subsidies in year 2021 at US$635 billion, of which more than 60% were coupled with production, and FAO (2021) estimates that, of the US$540 billion/year provided between 2013 and 2018 as net support to agricultural producers, about US$294 billion/year were price incentives, whereas US$245 billion/year were fiscal subsidies to farmers, with 70% tied to the production of a specific commodity. Importantly, agriculture subsidies are highly concentrated in a few large economies (i.e. China, India, USA, and the European Union) (OECD 2023), mostly benefit temperate products, and favour large farms and wealthy farmers (Damania et al. 2023).

Most subsidies to agriculture are considered environmentally harmful. Agriculture subsidies incentivizing intensification lead to excessive use of fertilizers, responsible for up to 17% of all nitrogen pollution in water in the past 30 years (Damania et al. 2023). At the same time, agriculture subsidies that incentivize extensification lead to land-use change with, for example, subsidies to soybeans, palm oil, and beef accounting for 14% of forest loss each year (Damania et al. 2023). Agriculture is also supported through irrigation subsidies for which we do not have global estimates despite their impacts on freshwater cycles (Koplow and Steenblik 2024). Moreover, agriculture subsidies have telecoupled effects. For example, livestock subsidies in the US increase soybeans demand, a major driver of deforestation in Brazil (Damania et al. 2023), while also enabling artificially low prices of key commodities, which negatively affects competitiveness of non-subsidized farmers in the Global South (Murphy and Hansen-Kuhn 2020).

Beyond explicit subsidies, globally, agriculture has large environmental externalities, and particularly greenhouse gas emissions, land degradation, water scarcity, and biodiversity loss. Estimations of the externalities generated by the global agriculture systems range enormously depending on the source, between US$3.3 trillion/year (FOLU 2019) and US$10 trillion/year (FAO 2023). FOLU (2019) estimates that deforestation, water scarcity, and land degradation linked to the global agricultural production costs around US$1.7 trillion/year, and agriculture emits up to 22% of total greenhouse gas emissions (IPCC 2023), with an estimated cost of US$1.45 trillion/year (FOLU 2019). When adding the costs associated with dietary patterns that contribute to diseases and reduce labour productivity, these costs reach 2021 US$12.7 trillion (FAO 2023).

### Fossil fuels

Fossil fuel extraction and consumption contribute to environmental degradation both directly through ecosystem degradation (e.g. DiSilvestro and Irvine-Broque 2023; Pellegrini et al. 2024) and indirectly through increased greenhouse gas emissions (Coady et al. 2017, 2019; Pörtner et al. 2021; BIOFIN 2024). Biodiversity hotspots, richness centres of endemic species, natural protected areas, urban areas, and the territories of indigenous people in voluntary isolation coincide with 609 gigabarrels of oil (Pellegrini et al. 2024). These socio-ecologically sensitive areas could be preserved, if kept off-limits to oil extraction. Fossil fuels combustion also harms nature through changes in the climate system driven by the release of greenhouse gases that include hydrocarbons, carbon and sulphur oxides, and air pollutants (Hassan et al. 2021; IPCC 2023; Kolawole and Iyiola 2023).

Despite its known perverse impacts, the extraction, production, refining, and consumption of coal, oil, and natural gas are heavily subsidized through financial and economic incentives (e.g. tax breaks, reduced royalty rates). Globally, governments invest between US$577 billion/year (Damania et al. 2023) and US$1.26 trillion/year (Black et al. 2023) to lower the price of fossil fuels (Fig. 2). Moreover, explicit subsidies to fossil fuels are on the rise, from US$0.5 trillion in 2020 to US$1.3 trillion in 2022, with sharply higher international fossil fuel prices (Black et al. 2023). Koplow and Steenblik (2024) estimated that fossil fuel subsidies reached a peak of US$1.5 trillion in 2022 before declining in 2023, underscoring their sensitivity to shifts in macroeconomic conditions. Emerging and developing countries account for over 75% of global fossil fuel subsidies (Coady et al. 2017; Solarin 2020), 97% of them being given to consumers (Black et al. 2023). Of the fossil fuel subsidies, by-product, undercharging for oil products, accounts for nearly 50%, coal another 30%, and natural gas nearly 20% (Black et al. 2023).

Fossil fuels also have high externalities, from health impacts of air pollution to global climate change. Externalities of the fossil fuel sector are estimated at about US$5.25 trillion/year (Black et al. 2023) (see Clements et al. 2013 and Coady et al. 2017 for similar estimates), of which externalizing local air pollution and climate change accounts for about 60% and undercharging for broader externalities and supply costs another 35% (Black et al. 2023).

It has been estimated that a 10% increase in fossil fuel subsidies per capita results in an increase in ecological footprint from 0.3 to 1.5% (Solarin 2020), whereas removing explicit fuel subsidies and internalizing externalities through corrective taxes would reduce global carbon dioxide (CO_2_) emissions by 43% below “business as usual” levels in 2030 (Black et al. 2023). According to modelling work, by 2030, full fuel price reform would also raise revenues of US$4.4 trillion, 3.6% of global GDP and avert about 1.6 million premature deaths per year from local air pollution (Black et al. 2023).

### Forestry

The use of forest lands for logging and other purposes (e.g. agriculture expansion, livestock ranching, road construction) generally leads to forest degradation and deforestation creating adverse environmental impacts (Giam 2017; Smith et al. 2023). Such impacts include increased soil erosion (Borrelli et al. 2017), increased greenhouse gas emissions (UNEP 2024), disruption of water cycle (Domínguez-Tuda and Gutiérrez-Jurado 2024), disrupted livelihoods (Oldekop et al. 2020), and decline or loss of biodiversity (Betts et al. 2017). For example, deforestation leads to breakdown of ecosystem functions and biodiversity loss (Betts et al. 2017; López-Bedoya et al. 2022). Deforestation not only affects above ground biodiversity, but it also affects soil biodiversity and reduces nutrient cycling and carbon storage in soils (Qu et al. 2024). Globally, the gross deforestation reached 6.37 million hectares in 2023 and was about 45% higher than what is needed to eliminate deforestation by 2030 (Forest Declaration Assessment Partners 2024). It is estimated that 3.7 million hectares of primary tropical forests were lost in 2023, while about 62.6 million hectares of forests were degraded in the same year (Forest Declaration Assessment Partners 2024).

Despite the negative impacts of forest loss on people and nature, subsidies that directly or indirectly encourage forest loss continue. There is no global compilation of forestry subsidies, but available estimates indicate that, in 2019, forestry subsidies from governments were about US$55 billion/year (Deutz et al. 2020). The latest estimates from Earth track suggest that the forestry subsidy increased to US$175 billion in 2024 (Koplow and Steenblik 2024) (Fig. 2). Forestry subsidies are provided at different stages of the forest conversion process, from land access and clearing to harvesting, processing, distribution, and consumption, and usually take the form of direct spending, concessional loans, tax exemptions, and—occasionally—regulatory and information instruments (McFarland et al. 2015).

Forestry subsidies contribute to forest loss and conversion and increase illegal logging (McFarland et al. 2015). For example, forestry subsidies have promoted harvesting old growth forests in Canada, processing imported logs—from Siberia and Canada—by sawmills in Japan, converting old growth forests into palm oil or forestry plantations in Indonesia, and converting forests to agricultural expansion in Brazil (Sizer 2000; McFarland et al. 2015). Subsidies that promote agricultural production are considered to have caused 14% of global deforestation (Damania et al. 2023).

Externalities of the forestry sector include the loss of ecosystem regulation and carbon services (Giam 2017; Smith et al. 2023). Estimations of externalities of the forestry sector are rare and there is large variation in valuation methods across estimates. For example, Interpol (2020) uses the value of illegal timber trade as a measurement approach, while the World Bank uses ecosystem services and/or lost tax revenue (Miranda Montero et al. 2019). Consequently, variation in available estimations of the externalities of the forestry sector is high, ranging between US$935 billion/year and US$1 930 billion/year (Miranda Montero et al. 2019; Koplow and Steenblik 2022). Notwithstanding the variation on these estimates, they represent a significant cost of inaction to curb subsidies that support or encourage or facilitate deforestation and forest degradation.

### Infrastructures

The development and maintenance of infrastructure (e.g. transport, electricity, water, telecommunications), and particularly of road and irrigation infrastructures, is also a known driver of nature decline. The construction of new roads disrupts ecological processes and leads to deforestation, habitat loss, watershed damage, and light, noise, and chemical pollution, being particularly impactful when connecting remote locations (Koplow and Steenblik 2022). Globally, irrigation is the largest consumptive use of water, accounting for 70 to 90% of total water use in developing countries and for more than 30% in OECD countries (Malik 2008). Irrigation subsidies are considered environmentally harmful because they discourage efficient water use, resulting in surface and groundwater depletion and/or degradation, changes in water flow, loss of freshwater habitats, pollution, and salinity (Barbier 2019).

The development and maintenance of infrastructure, in general, is heavily subsidized, reaching about US$2.3 trillion in 2015, or 12% of global GDP (Oxford Economics 2017). Subsidies to develop and maintain roads include exemptions on property taxes for land designated for roads, insufficient user fees for state roads, or under-pricing road-damaging loads. Global data on roads subsidies are extremely sparse and mainly documented as a share of GDP (Foster et al. 2022) or confounded with expenditures across different types of infrastructures (Fay et al. 2019). Estimates of subsidies to road construction and maintenance range from an average of US$224 billion/year between 2015 and 2019 (ITF Transport Statistics 2021) to US$586 billion/year for the period 2007–2025 (Oxford Economics 2017).

Irrigation subsidies include the development, rehabilitation, modernization, operation, and maintenance of irrigation and drainage infrastructure, but also setting water prices below cost, assisting pumping costs, clearing titles to water rights, and socializing water treatment costs (Ward 2010; Barbier 2019; Hellegers et al. 2022). Global estimates of irrigation subsidies are difficult to obtain as they are often confounded with estimates of other infrastructures (Fay et al. 2019), agriculture services (FAO et al. 2021) or water subsidies (including access to drinking water and sanitation) (FAO et al. 2021). A conservative figure, based on data from 38 countries, puts irrigation subsidies at US$5.82 billion/year during 2015–2019, or US$195 year/irrigated hectare (Damania et al. 2023). More global estimates of irrigation subsidies range from US$30 billion (Kjellingbro and Skotte 2005) to US$158 billion in 2015 (Oxford Economics 2017). The distribution of irrigation subsidies is highly skewed and uneven, being disproportionally high in high- and upper-middle-income countries and largely benefiting higher income groups or those holding lands (Gany et al. 2019).

There is currently no estimate of the cost of externalities associated with road infrastructures available or under construction. Externalities associated with irrigation infrastructure expansion include the extraction of non-renewable groundwater, water pollution, and changes in seasonality of water flows and sedimentation patterns (Tietenberg and Lewis 2014; Hellegers et al. 2022), but no calculation of the cost of externalities associated with irrigation is available.

### Fisheries and aquaculture

Subsidies to the fisheries sector can distort markets and contribute to unfair trade practices, hinder international fisheries cooperation, exacerbate inequality by undermining the viability of small-scale producers, lead to higher CO_2_ emissions, act as a driver for illegal, unreported and unregulated (IUU) fishing, and increase the risk of overfishing by increasing fishing fleet capacity (Villasante et al. 2022; Skerritt et al. 2023).

Global estimates of explicit subsidies to the fishing sector in 2018 were of US$35.4 billion/year, of which US$22.2 billion/year (or 63%) were capacity-enhancing subsidies (Sumaila et al. 2019) (Fig. 2). Koplow and Steenblik (2024) estimate fisheries subsidies at US$55 billion in 2023.

Subsidies to the fisheries and aquaculture sector include management support and assistance, access agreements, infrastructure development, and support for fuel and business development (Matthews and Karousakis 2022). Fisheries subsidies have encouraged IUU fishing, resulting in overfishing and marine biodiversity decline and a higher risk of food security of billions of coastal livelihoods (Sumaila et al. 2019; Villasante et al. 2022). More than half of high seas fishing activities, which contribute the most to CO_2_ emissions (Mariani et al. 2020) and only marginally to global food security (Schiller et al. 2018), would not be profitable without subsidies and low labour costs (Sala et al. 2018). For instance, subsidies to high seas bottom trawlers amount to approximately US$152 million annually, accounting for 25% of their total landed value. However, this fleet typically achieves profits of only about 10% of their landed value, indicating that many bottom trawlers would not be economically sustainable without subsidies (Sumaila et al. 2010a, b). Most marine fisheries subsidies are provided by China, the EU, USA, and the Republic of Korea (Schuhbauer et al. 2020; Skerritt et al. 2023), all particularly active in distant waters of countries in the Global South (namely in West Africa and South America) and high seas fishing (Villasante et al. 2014).

Countries with subsidized fisheries have an unfair advantage in exploiting the ocean (Sumaila et al. 2010a, b; Schuhbauer et al. 2020; Sumaila et al. 2024). For example, the total factor productivity of the EU small-scale fishing fleet, one of the largest in the world, is almost twofold in the North Atlantic and 16% higher in the Mediterranean and Black seas, compared to large-scale vessels (Villasante et al. 2022). Fishing activities outside national jurisdictions receive between 20 and 37% of total fisheries subsidies (Sumaila et al. 2024) and have a disproportionately high negative impact on nations with low or very low Human Development Index, low management capacity, vulnerable fish stocks, and active coastal communities (Skerritt et al. 2023).

The lack of effective regulations to reduce overcapacity and prevent overfishing produces large externalities, with foregone economic benefits estimated at US$83 billion/year, or nearly 20% of the size of the sector (World Bank 2017).

The expansion of aquaculture through harmful subsidies can contribute to a higher competition for space and resources with small-scale fisheries, leading to tensions over water quality, feed sources, and habitat (Roberts et al. 2024). Aquaculture also needs pelagic species such as anchovies and sardines as fishmeal and oil to grow (Sumaila et al. 2022), which are the species with the highest nutrition value for human consumption (Golden et al. 2016). Nevertheless, aquaculture has been the fastest growing animal food production sector in recent decades (Naylor et al. 2023).

Aquaculture is heavily subsidized, with subsidies aiming to strengthen technological development, innovation, and knowledge transfer to enhance the competitiveness and viability of aquaculture enterprises, protect and restore aquatic biodiversity, boost ecosystems related to aquaculture, and promote resource-efficient systems. During the 2007–14 period, EU structural funds invested in aquaculture reached €1.17 billion, representing 2.5% of the value of each kg of fish farmed in the EU (Guillen et al. 2019). China, Norway, Chile, and Indonesia represent about 70% of the world’s mariculture (FAO 2020), providing about US$5.24 billion/year in direct subsidies to aquaculture, a figure that excludes indirect subsidies such as tax exemptions and insurance premiums (Mejaes 2021).

### Metal mining

Metal extraction, beneficiation, smelting, and refining have both local and global environmental impacts. Metal mining affects extraction sites, with impacts that range from habitat loss to soil, air, surface and ground water contamination (Murguía et al. 2016; Northey et al. 2017; Sonter et al. 2018; Agboola et al. 2020). Metal mining also contributes to toxic waste generation and land subsidence (Mudd 2009; Nuss and Eckelman 2014). Metal mining impacts are particularly concentrated in vulnerable ecosystems, with about 80% of global metal ore extraction in 2019 originating from five of the six most species-rich biomes and taking place at 20km or less from protected territories (Luckeneder et al. 2021). In parallel to the increase in tax incentives to metal mining since the late 1990s (IGF 2019), mining volumes have doubled in tropical moist forest ecosystems, with 90% of all extraction sites occurring in relative water-scarce areas (Luckeneder et al. 2021). The trend is expected to continue, with demand for critical minerals (e.g. copper, lithium, nickel, cobalt, and rare earth elements) essential for electronics, renewable energy systems, batteries, and defence applications quickly growing (IEA 2023).

Subsidies to the metal mining sector include subsidies for the extraction (e.g. tax exemptions, public provision of finance on concessionary terms, public provision of services (e.g. geoscientific information) at below cost recovery, advantageous import and export tariffs, and consumption of metal minerals (e.g. export restrictions to support domestic industries) (McCarthy and Börkey 2018). Metal mining subsidies are typically received by producers, granted through law, and stabilized for about 20 years (McCarthy and Börkey 2018; IGF 2019). Subsidies are more common for primary than for secondary metals (i.e. metals produced from scrap) (McCarthy and Börkey 2018) despite the considerably higher environmental impacts of primary metals (OECD 2019).

Until recently, there were no global data available on metal mining subsidies. A recent report (Koplow and Steenblik 2024) estimates that subsidies to non-energy mining correspond to about US$ 40 billion; this is a conservative estimate giving that mining activities remain largely untracked, the scope and scale of metal mining being poorly documented, particularly for illegal gold and diamond mining (Koplow and Steenblik 2024). Most available data on metal mining subsidies refer to specific countries (Koplow 1994; Scharf 1999; Griffith 2013; Johansson et al. 2014), specific support measures (Lambrechts et al. 2009; Fogarty and Sagerer 2016), or specific metals (OECD 2015). The scarce available information shows that metal mining subsidies amount to billions of dollars for single countries (e.g. Griffith 2013; McCarthy and Börkey 2018). For example, in 2010, the Swedish Government granted metal mining subsidies for an estimated amount of €4 billion (Johansson et al. 2014), and subsidies to the Chinese domestic steel sector between 2000 and 2007 amounted to US$27 billion (Haley and Haley 2013). Metal mining subsidies are most common in emerging countries endowed with mineral resources (IGF 2019), although they are also present in developed countries. For example, in the US, the Inflation Reduction Act established US$500 million in incentives for critical minerals mining and processing, and, in 2022, Australia lend US$1.05 billion to build a fully integrated rare earths separation facility and directed part of its US$15 billion National Reconstruction Fund to critical minerals companies that build national processing, refining or manufacturing capacity (PwC 2023).

The cost of inaction in metal mining, especially given the anticipated rise in demand, is projected to result in significant direct and indirect environmental, health, and societal impacts (see Agboola et al. 2020 for a discussion of health impacts associated with metal mining in specific regions). Yet there is significant potential for transformative change in the metal mining sector given the high recyclability of most metals, which renders this sector a prime candidate for shifts towards a more circular economy (McCarthy and Borkey 2018). A study of e-waste processing in China finds that urban e-waste metal mining is now recovered at costs similar to virgin mining of ore (Zeng et al. 2018), thus highlighting the high potential for a shift to reuse and recycling in this sector.

## Future directions

Overall, the data compiled suggest that subsidies to six economic sectors directly driving nature decline range from US$1.7 and US$3.5 trillion annually, which represents between 1.6 and 3.3% of the global GDP in 2023 (using an estimate of US$105.4 trillion). The range comprises Koplow and Steenblik (2024) estimate of global subsidies to be US$2.7 trillion in 2023 (or 2.6% of global GDP). For comparison, subsidies for clean technology deployment are in the order of US$100 billion (Taylor 2020). Estimates of environmental externalities associated with four of these sectors range between US$10.5 trillion/year and US$22.6 trillion/year (or between 10 and 21.4% of the 2023 global GDP), with agriculture and fossil fuels, the two sectors most heavily subsidized, generating the largest externalities.

These figures only provide a conservative estimate of the magnitude of subsidies and environmental externalities of economic sectors. Data gaps and measurement problems prevent having a complete picture of the costs of subsidies and externalities. Data gaps affect both the measurement of subsidies and externalities, although more efforts have been made to measure subsidies than to assess externalities. All sources used for our estimations acknowledge data gaps in compiling information on public subsidies. For some sectors, there is no information available for some countries or regions. For example, recent global data are available for the mining sector but are extremely conservative given the lack of tracking in this sector (Koplow and Steenblik 2024). Other sectors, e.g. plastics, construction, are only recently starting to be tracked. Data gaps are likely to continue while reporting of subsidies remain voluntary.

Beyond data gaps, measurement problems also affect the calculation of costs of subsidies and externalities across different economic sectors. For example, available data on the costs of subsidies are based on different valuation methods, making aggregation irrelevant and comparisons difficult (Coady et al. 2017; Matthews and Karousakis 2022). The quantification of environmental externalities from sectors directly driving nature decline has received even less attention. We found no global estimates of externalities related to the infrastructure and mining sectors—two areas where subsidy data are also lacking. Moreover, efforts to quantify externalities face significant methodological challenges, primarily due to the complexities in valuing impacts on ecosystems. For example, many environmental externalities manifest over long timeframes or accumulate gradually, making them challenging to quantify in the short term. Additionally, the cumulative and indirect effects of these externalities are difficult to model, given the numerous interactions and feedback loops involved. Beyond environmental impacts, many sectors also generate health-related externalities, which are often overlooked in existing studies. For example, estimates of the significant externalities in agriculture seldom account for costs such as child stunting, pesticide exposure, land degradation, antimicrobial resistance, or illness from unsafe food (FAO 2023). When they are included (e.g. FOLU 2019), there is often little explanation of how these costs are quantified or how they translate into increased health costs. In sum, not only there are insufficient data to establish a baseline for assessing the magnitude of subsidies and externalities, but also a lack of universally accepted frameworks for their quantification. This results in inconsistencies across methodologies and makes comparisons difficult between regions, sectors, or studies. Without a standardized approach, it is impossible to effectively assess and monitor the impacts of potential reforms.

Our efforts also suggest that in addition to filling data gaps and harmonizing methodological approaches, the creation of a baseline to account for the costs of subsidies and externalities of economic activities driving nature decline would require differentiating between environmentally harmful subsidies and subsides directed to support activities that protect or restore nature. Examples exist of repurposing harmful subsidies to provide financial and economic resources both to halt and reduce biodiversity loss from ongoing activities as well as to reallocate capital towards activities that are nature restoring and equitable. For example, New Zealand reformed its fisheries subsidies and offers no subsidy to its fishing industry, but all subsidy funds use strict sustainability criteria as a condition for access (Campling and Havice 2013). England’s Environmental Land Management Scheme is designed to financially reward sustainable farmland management for the generation of public goods (Hurley et al. 2022). Zambia has also developed an inventory of possible financing options to enhance biodiversity, including scaling back conventional agriculture subsidies and reallocating funds to climate smart agriculture and biodiversity conservation, introducing a penalty to agricultural subsidy recipients who purchase inputs harmful to biodiversity, and using biodiversity revenues (e.g. national parks entries and trophy hunting licences) and environmental fiscal measures (e.g. carbon tax, forestry revenue) to finance a National Biodiversity Conservation Fund (Mweemba 2018). If countries actively work towards the CBD target of “reduction of harmful subsidies by at least US$500 billion per year and scale up positive incentives for biodiversity by 2030”, the volume and scale of subsidies directed towards activities that protect or restore nature are likely to increase. This underscores the importance of documenting the direction and allocation of these subsidies.

A baseline account of the costs of subsidies and externalities of economic activities driving nature decline would allow to assess the efficiency of public expenditures. For example, a study focusing on fossil fuel subsidies in South Africa shows that monetary revenues associated with fossil fuel subsidies were considerably lower than social costs linked to health and climate (Bridle et al. 2022). Understanding such costs is important. Moreover, such baseline would also allow monitoring the multiple impacts and trade-offs that manifest at different temporal and spatial scales. Subsidies and their reforms can have multiple impacts that need to be monitored. For example, the removal of support to industrial agriculture, while potentially resulting in nature preservation and reduction of greenhouse gas emissions in the long-term, might lead to higher food costs and hurt farm incomes in the short term (Pörtner et al. 2021), as seen in the internal food distribution crisis in Zambia resulting from a post-structural adjustment withdrawal of support for government-funded institutions in charge of food distribution (Seshamani 1998).

The distribution of subsidies and environmental externalities involves spatial trade-offs that extend beyond national borders, as long and complicated supply chains result in cross-border impacts. For example, removing subsidies to maize or soybean cultivation in the USA might result in higher global prices, thus affecting importing countries and encouraging conversion of grasslands or forests to cropland in other regions (Stiglitz and Charlton 2005; Searchinger et al. 2018). Contrary, in some other instances, the removal of subsidies in one country can have telecoupled benefits. For example, a removal of subsidies for distant water fishing fleets could improve food and nutrition security in low-income countries reliant on fishing for food and nutrition security (Tickler et al. 2018; Skerritt et al. 2023; Sumaila et al. 2024; Sumaila 2024; Teh et al. 2024). A baseline account of the costs of subsidies and externalities of economic activities driving nature decline would allow to monitor such multiple and full impacts and trade-offs.

A better understanding of the complexity, size, design, and effects of subsidies and externalities of economic sectors contributing to environmental degradation could facilitate and expedite discussions to strengthen the implementation of multilateral agreements. This is an essential aspect of the global economic system that needs to be transformed if a more sustainable and equitable future for the planet and people is to emerge.

## Supplementary Information

Below is the link to the electronic supplementary material.

Supplementary file1 (PDF 243 kb)
