# Correction: The costs of subsidies and externalities of economic activities driving nature decline

**DOI:** 10.1007/s13280-025-02310-w

**Published:** 2025-12-19

**Authors:** Victoria Reyes-García, Sebastian Villasante, Karina Benessaiah, Ram Pandit, Arun Agrawal, Joachim Claudet, Lucas A. Garibaldi, Mulako Kabisa, Laura Pereira, Yves Zinngrebe

**Affiliations:** 1 Institut de Ciència i Tecnologia Ambientals, Universitat Autònoma de Barcelona (ICTA-UAB), Cerdanyola del Vallès, 08193 Barcelona, Spain; 2 Institució Catalana de Recerca i Estudis Avançats (ICREA), 08010 Barcelona, Spain; 3 Departament d’Antropologia Social i Cultural, Universitat Autònoma de Barcelona, Cerdanyola del Valles, 08193 Barcelona, Spain; 4 CRETUS, EqualSea Lab, Department of Applied Economics, University de Santiago de Compostela, 15782 A Coruña, Spain; 5 Xunta de Galicia, Spain; 6 Center for Cross-Disciplinary Research in Environmental Technologies (CRETUS), Av Rua Constantino s/n, 15782 Santiago de Compostela, Spain; 7 Department of Geography, Environment and Geomatics, University of Guelph, Guelph, ON Canada; 8 Centre for Environmental Economics and Policy, UWA School of Agriculture and Environment, The University of Western Australia, 35 Striling Highway, Crawley, Perth, WA 6009 Australia; 9 Global Center for Food, Land and Water Resources, Research Faculty of Agriculture, Hokkaido University, Kita 9, Nishi 10, Kita-ku, Sapporo, Hokkaido 060-8589 Japan; 10 The Western Australian Biodiversity Science Institute (WABSI), Perth, Australia; 11 School for Environment and Sustainability, University of Michigan, Ann Arbor, USA; 12 Keough School of Global Affairs, University of Notre Dame, Notre Dame, IN USA; 13 National Center for Scientific Research, PSL Université Paris, CRIOBE, Maison de l’Océan, 195 Rue Saint-Jacques, 75005 Paris, France; 14 Instituto de Investigaciones en Recursos Naturales, Agroecología y Desarrollo Rural (IRNAD), Universidad Nacional de Río Negro, Mitre 630, 8400 San Carlos de Bariloche, Río Negro Argentina; 15 Consejo Nacional de Investigaciones Científicas y Técnicas, San Carlos de Bariloche, Río Negro Argentina; 16 Global Change Institute, University of the Witwatersrand, Private Bag 3, WITS, Johannesburg, 2050 South Africa; 17 Stockholm Resilience Centre, Stockholm University, Stockholm, Sweden; 18 Johannesburg, South Africa; 19 Helmholtz Centre for Environmental Research – UFZ, Permoserstr 15, 04318 Leipzig, Germany

**Correction to Ambio (2025) 54:1128–1141** 10.1007/s13280-025-02147-3

In this article, the authors identified errors in Fig. 2 related to the units displayed. These errors arose from confusion between the meanings of “billion” and “trillion” in English and their equivalents in French and Spanish, the lead author native language, which use a different numerical convention (where un billion/un billón = 1012 rather than 10^9^). The correct data was reported in the text. In addition, some bars representing missing data may have caused confusion. The incorrect and corrected version of Fig. 2, along with the updated caption, are provided in this correction.

Incorrect version of Fig. 2:

**Fig. 2:**
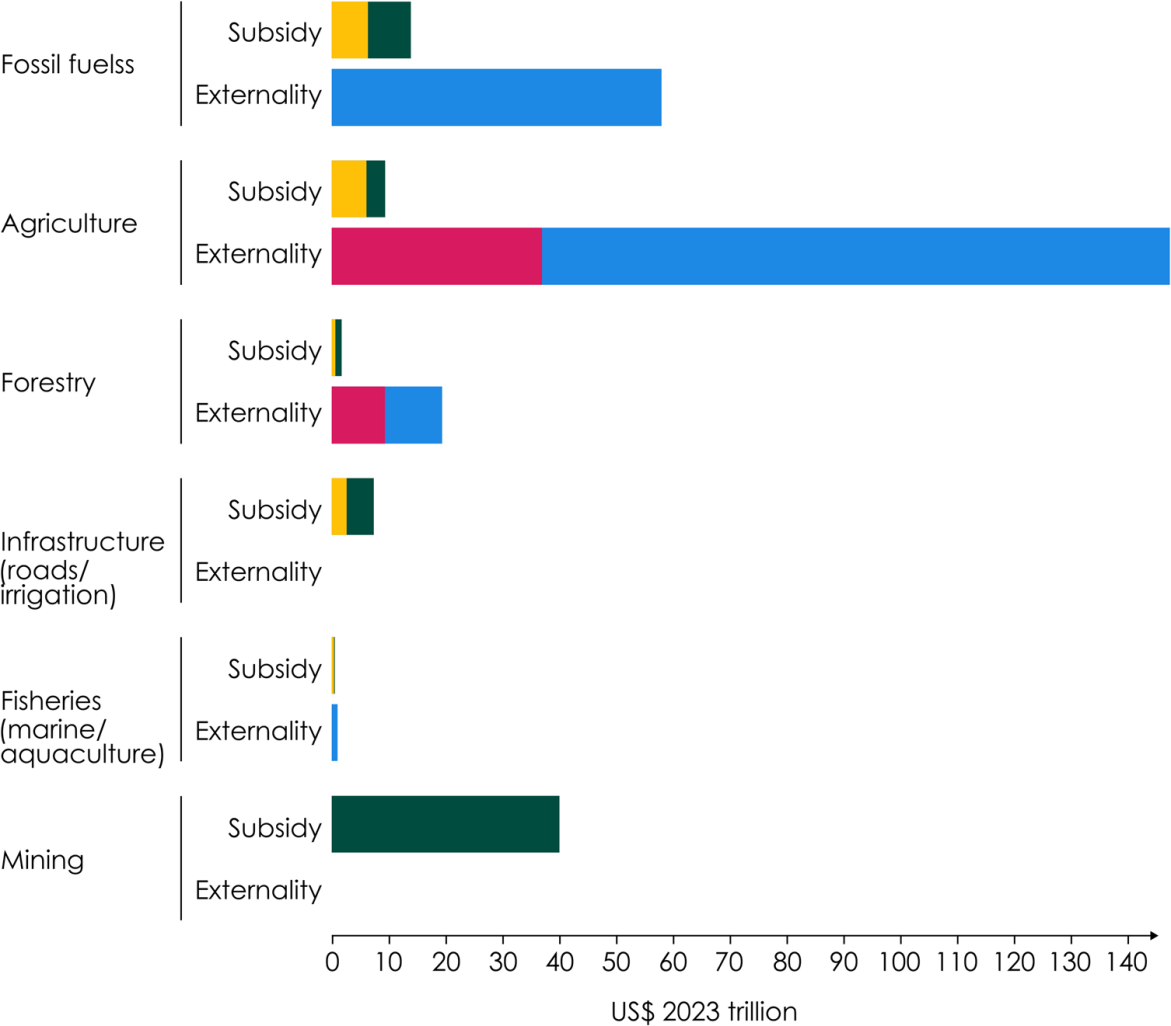
Bar chart showing the cost of subsidies and externalities of six economic sectors, in US$ trillion

Correct version of Fig. 2:

**Fig. 2:**
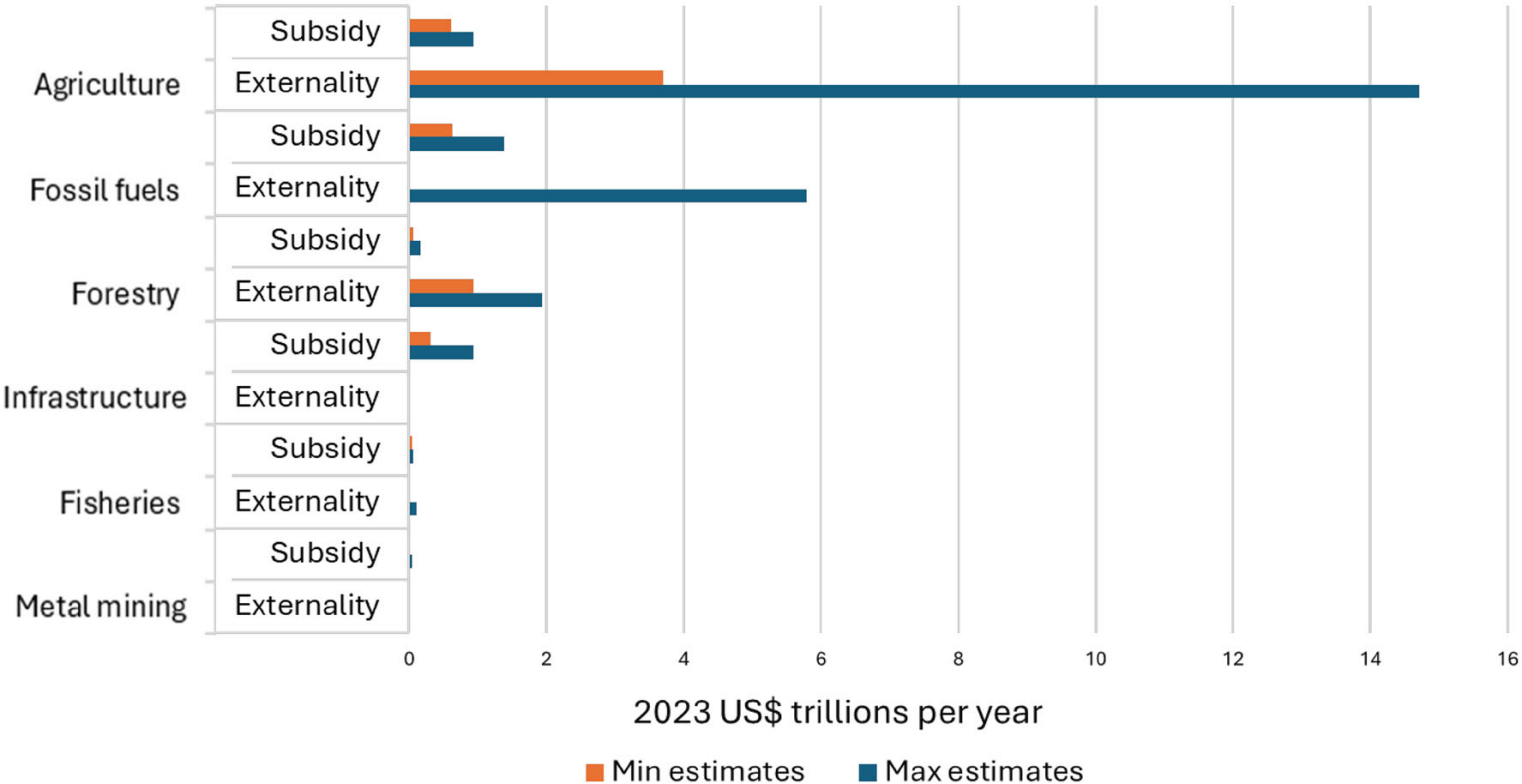
Bar chart showing estimated annual costs of subsidies and externalities for six economic sectors, expressed in 2023 US$. To construct the figure, estimates drawn from different sources and years were converted to 2023 US$ using inflation adjustments based on the U.S. Consumer Price Index for the corresponding data years (see Supplementary file1 10.1007/s13280-025-02310-w)

The original article has been corrected.

