# Supplementary material for: The costs of subsidies and externalities of economic activities driving nature decline
Source: Ambio. 2025 Feb 28;54(7):1128–41. doi: 10.1007/s13280-025-02147-3 (PMC12133627; doi:10.1007/s13280-025-02147-3)
Supplement: Supplementary file 1 — Supplementary file1 (PDF 243 kb) [file 13280_2025_2147_MOESM1_ESM.pdf]

Ambio

Supplementary Information

This supplementary information has not been peer reviewed.

Title: **The costs of subsidies and externalities of economic activities driving nature decline**

Authors: Reyes-García, V., S. Villasante, K. Benessaiah, R. Pandit, A. Agrawal, J. Claudet, L.A. Garibaldi, M. Kabisa, L. Pereira, Y. Zinngrebe

Table S1

Databases reporting subsidies

Database	Economic sector	Geographical scope	Information covered	Source
EU – State Aid	Any subsidy	EU-27	State aid cases, year, description	Link
Fossil Fuel Subsidy Tracker	Fossil Fuel	Any country	Data on subsidy by year, fuel, and country from all major international databases including the OECD, IMF, and IEA.	Link
IEA – Fossil Fuel Subsidies Database	Fossil fuel	IEA members	Country, year, status, jurisdiction, description containing aim and reforms if any	Link
IMF – Fossil Fuel Subsidies Data	Energy	Global	General information, beneficiaries, magnitude, impacts, benefits of reform, plan for reform. Includes time series data on subsidies and efficient fuel prices at the country-level.	Link
Joint Subsidy Platform (JSP)	Agriculture, Fossil fuels, Fisheries, cross-sectoral	Agriculture – OECD, EU, and 11 emerging economies.<br>Fossil fuels - 51 OECD, G20, and EU Eastern Partnership.<br>Fisheries - 40 OECD, large fishing nations. Cross-sectoral -	Information on subsidies collected by the International Monetary Fund (IMF), Organisation for Economic Co-operation and Development (OECD), World Bank Group (WBG), and World Trade Organization (WTO)	Link

		reporting countries of the Government Finance Statistics Manual 2014		
OECD – Government Support and Subsidies	Agriculture, fisheries, industrial, fossil fuels	EU-27, except BG, CY, HR, MT, RO	Country, type of subsidies, characteristics, activities supported, annual cost target groups.	Link
World Bank – Subsidies and other transfers	Any subsidy	Any countries	Subsidies, grants, and other social benefits to private and public enterprises; grants to foreign governments, international organizations, and other government units; and social security, social assistance benefits, and employer social benefits in cash and in kind.	Link
WTO – Subsidy Database	Any subsidy	WTO members	Description, policy objective, duration and type of measure, as well as information on the eligibility criteria, monetary amounts and statistical data on the potential trade effects of the measure.	Link

Table S2

Data sources for Figure 2

Economic sector	Cost estimated	Extend	US$ billion	Year	Adjusted 2023 US$ billions	Source
Fossil fuels	Subsidy (Min)	Global consumption	577	2021	637	(Damania et al., 2023)
	Subsidy (Max)	Global	1260	2021	1390	(Black et al., 2023)
	Externality	Global	5250	2021	5792	(Black et al., 2023)
Agriculture	Subsidy (Min)	Global	610	20 23	610	(Koplow & Steenblik, 2024)
	Subsidy (Max)	OECD countries	851	2021	939	(OECD, 2023)
	Externality (Min)	Global	3100	2018	3693	(FOLU, 2019)
	Externality (Max)	Global	12 700	2020	14 718	(FAO, 2023)
Forestry	Subsidy (Min)	Global	55	2019	64	(Deutz et al., 2020)
	Subsidy (Max)	Global	175	2023	175	(Koplow & Steenblik, 2024)
	Externality (Min)	Global	935	2023	935	(Koplow & Steenblik, 2024)
	Externality (Max)	Global	1939	2023	1939	(Koplow & Steenblik, 2024)
Infrastructure (Road / irrigation)	Subsidy (Min)	Global	224 / 30	2019 / 2004	263 / 48	(ITF Transport Statistics, 2021; Kjellingbro & Skotte, 2005)
	Subsidy (Max)	Global	586 / 158	2015 / 2015	739 / 199	(Oxford Economics, 2017)
Fisheries (Marine / aquaculture)	Subsidy (Min)	Global	35.4 / 5.2	2018	42 / 6	(Mejaes, 2021; Skerritt et al., 2023)
	Subsidy (Max)	Global	55/ 5.2	2023 /2018	55 / 6	(Mejaes, 2021; Skerritt et al., 2023)
	Externality	Global (marine)	83	2016	103	(World Bank, 2017)
Metal mining	Subsidy	Global	40	2023	40	(Koplow & Steenblik, 2024)

To adjust the estimated values to 2023 USD billions, we apply inflation correction using the U.S. Consumer Price Index for year of data (https://www.bls.gov/cpi/tables/).

References for Supplementary Materials

Black, S., Liu, A., Parry, I., & Vernon, N. (2023). *IMF Fossil Fuel Subsidies Data: 2023 Update.*

Damania, R., Balseca, E., de Fontaubert, C., Gill, J., Kim, K., Rentschler, J., Russ, J., & Zaveri, E. (2023). Detox Development: Repurposing Environmentally Harmful Subsidies. *Detox Development*. https://doi.org/10.1596/978-1-4648-1916-2

Deutz, A., Heal, G. M., Niu, R., Swanson, E., Townshend, T., Zhu, L., Delmar, A., Meghji, A., Sethi, S. A., & Tobin-de la Puente, J. (2020). *Financing Nature: Closing the global biodiversity financing gap*.

FAO. (2023). *The State of Food and Agriculture 2023 – Revealing the true cost of food to transform agrifood systems*. https://doi.org/https://doi.org/10.4060/cc7724en

FOLU. (2019). *Growing Better: Ten Critical Transitions to Transform Food and Land Use*.

ITF Transport Statistics. (2021). *Transport infrastructure investment and maintenance*. OECD ILibrary. https://www.oecd-ilibrary.org/finance-and-investment/data/itf-transport-statistics/transport-infrastructure-investment-and-maintenance_g2g55573-en

Kjellingbro, P., & Skotte, M. (2005). *Environmentally harmful subsidies: linkages between subsides, the environment and the economy*.

Koplow, D., & Steenblik, R. (2024). *Protecting Nature by Reforming Environmentally Harmful Subsidies: An Update*. https://www.earthtrack.net/document/protecting-nature-reforming-environmentally-harmful-subsidies-update

Mejaes, B. A. (2021). *Classifying and estimating aquaculture subsidies and their risks to the marine environment*. https://doi.org/10.14288/1.0398098

OECD. (2023). *Agricultural Policy Monitoring and Evaluation* (Agricultural Policy Monitoring and Evaluation). OECD. https://doi.org/10.1787/22217371

Oxford Economics. (2017). *Global Infrastructure Outlook.*

Skerritt, D. J., Schuhbauer, A., Villasante, S., Cisneros-Montemayor, A. M., Bennett, N. J., Mallory, T. G., Lam, V. W. L., Arthur, R. I., Cheung, W. W. L., Teh, L. S. L., Roumbedakis, K., Palomares, M. L. D., & Sumaila, U. R. (2023). Mapping the unjust global distribution of harmful fisheries subsidies. *Marine Policy*, *152*, 105611. https://doi.org/10.1016/J.MARPOL.2023.105611

World Bank. (2017). The Sunken Billions Revisited: Progress and Challenges in Global Marine Fisheries. In *The Sunken Billions Revisited: Progress and Challenges in Global Marine Fisheries*. Washington, DC: World Bank. https://doi.org/10.1596/978-1-4648-0919-4
